# Neurological manifestations of COVID-19 and its vaccines: an updated comprehensive review with an insight into pathophysiology

**DOI:** 10.1097/MS9.0000000000003931

**Published:** 2025-09-18

**Authors:** Khabab Abbasher Hussien Mohamed Ahmed, Tagwa Kalool Fadlalla Ahmad, Mohamed Ismail Abdu Ismail, Ammar T. Elgadi, Esraa Hassan Salih Elhaj, Ghassan E. Mustafa Ahmed, Faheemullah Khan, Mohammed Babiker Habbiballah Mohammed, Gaffar Alemam A. Manhal, Ahmed Daffalla Mussaad Mohammed, Mustafa Mohamed Ibrahim Ali, Mohammed Elmustafa Adil Abdullah Mohammed, Mostafa Meshref, Abbasher Hussien

**Affiliations:** a Faculty of Medicine, University of Khartoum, Khartoum, Sudan; b Faculty of Medicine, Ahfad University for Women, Omdurman, Sudan; c Faculty of Medicine, Sudan International University, Khartoum, Sudan; d Cardiovascular Imaging Fellow, Diagnostic Institute, Cleveland Clinic, Main Campus, Ohio, USA; e Department of Neurology, Faculty of Medicine, Al-Azhar University, Cairo, Egypt

**Keywords:** COVID-19, COVID-19 pathophysiology, neurological manifestations, SARS-coV-2, vaccination

## Abstract

The COVID-19 pandemic caused by SARS-CoV-2 has had a significant global impact on the respiratory system and multiple organ systems, including the nervous system. Neurological manifestations associated with COVID-19 infection and its vaccines have been increasingly recognized, ranging from problems with smell and taste to more severe conditions such as encephalitis, stroke, and Guillain–Barré syndrome. This narrative review critically evaluates the neurological manifestations of COVID-19 infection and its vaccines, providing insights into potential pathophysiological mechanisms. A comprehensive literature search was conducted, and data were retrieved from various databases. The prevalence, types, and severity of neurological symptoms in COVID-19 patients were discussed. The possible mechanisms of neurological injury in COVID-19 were explored, including direct viral invasion, hypoxic brain injury, immune-mediated damage, and cerebrovascular injury. Furthermore, the review addressed the neurological complications associated with COVID-19 vaccination. While severe vaccine-related adverse effects remain rare, understanding their occurrence is essential for risk assessment and public health interventions. In conclusion, COVID-19 can affect the nervous system in various ways, leading to various neurological symptoms. Further research is necessary to enhance our understanding of these manifestations and develop effective preventive and treatment strategies to manage this global health crisis.

## Introduction

Severe acute respiratory syndrome coronavirus 2 (SARS-CoV-2) is a member of the Coronavirus family. It was first identified in Wuhan, China, in December 2019, leading to a global pandemic in March 2020^[1,2]^. The COVID-19 outbreak in Sudan represents a component of this global pandemic, leading to substantial morbidity and mortality^[3,4]^. The presence of the virus in Sudan was verified in March 2020^[5]^. The primary impact of the virus is on the respiratory system, causing pneumonia and acute respiratory distress syndrome (ARDS). However, it has also been found to affect various organs, including the kidneys, brain, heart, liver, and others, resulting in multi-organ dysfunction^[4]^. Additionally, severe complications such as cytokine storm, septic shock, and blood clotting abnormalities may occur^[6]^. In Sudan, we have seen cases of COVID-19-related neurological sequelae such as convulsions, meningitis, encephalitis, stroke, infarction, and Guillain–Barré syndrome (GBS), as well as relapses of multiple sclerosis after infection. The central and peripheral nervous systems are also affected by neurological symptoms and consequences^[7]^.

**HIGHLIGHTS**

- Broad Neurological Impact: COVID-19 affects both the central and peripheral nervous systems, manifesting as anosmia, ageusia, seizures, stroke, Guillain–Barré syndrome (GBS), and encephalitis, with some symptoms persisting into “long COVID.”
- Diverse Pathophysiological Mechanisms: Neurological injury in COVID-19 stems from direct viral invasion, cytokine storms, hypoxia, and cerebrovascular complications – often facilitated by disruption of the blood–brain barrier or immune-mediated pathways.
- Cranial Nerve and Long-Term Sequelae: Multiple cranial nerves, including the olfactory and facial nerves, are frequently affected. Long COVID cases reveal persistent neurological symptoms such as fatigue, confusion, paresthesia, and dizziness.
- Post-Vaccination Neurological Events: While generally rare and mild, some severe neurological adverse events post-COVID-19 vaccination have been reported – such as CVST, transverse myelitis, GBS, and stroke – especially with adenoviral vector vaccines.
- Risk-Benefit Justification: Despite reports of neurological complications from both COVID-19 infection and vaccines, the risk associated with vaccination remains significantly lower than that from the disease, reinforcing the importance of immunization.

Our comprehensive analysis indicated that COVID-19 can present with various neurological symptoms either during the initial phase or throughout the progression of the illness. However, it is crucial to gain a deeper understanding of these symptoms, to enhance our understanding and facilitate the development of preventive strategies to effectively manage this global health crisis^[8]^. Additionally, the neurological aspects of the disease, including altered mental status, seizures, and febrile convulsions^[9]^. Nevertheless, motor impairments have not received significant attention in research^[10]^. Therefore, it is crucial to conduct further investigations to document the prevalence and extent of these neurological manifestations.

Mild adverse reactions to COVID-19 vaccines may manifest as discomfort and swelling at the injection site, urticaria rashes, fever, and headaches. In some cases, more severe adverse reactions, including neurological manifestations, myocarditis, and autoimmune disorders, have been reported following mass vaccination, requiring hospitalization for certain individuals. When considering the neurological manifestations associated with the COVID-19 pandemic, it is essential to assess their prevalence and potential relationship to the vaccines^[11]^. Additionally, weighing the benefits of vaccination against the potential risks is crucial to make informed decisions regarding public health and individual well-being.

In the time of COVID-19 pandemic, many emerging reports suggested that the SARS-CoV-2 has inimical effects on the neurological functions and even causes serious neurological damage. The review focused on the manifestation of various neurological disorders linked with both the central nervous and peripheral nervous systems, the mechanism by which coronavirus can induce these diseases and the possible routes that this virus can use to reach the brain. We also investigate the possible pathogenesis behind the development of neurological manifestations after receiving a COVID-19 vaccine.

## Methods

This study is a narrative review of the neurological manifestations of COVID-19 and its vaccines. Two distinct search strategies were implemented: one for the neurological manifestations of COVID-19 and another for the neurological complications of COVID-19 vaccines.

Data used to prepare this narrative review were retrieved from MEDLINE (PubMed, Google Scholar, Scopus and Science-Direct search) using the search terms “coronavirus,” “SARS-COV,” “SARS-COV2,” “neurological manifestation,” “neurological deficits,” “vaccinations of COVID-19,” “Cranial nerves,” “neurological complications,” “neurological side effects,” and “COVID-19 vaccine’s side effect.” These terms were used individually and in various combinations to ensure a comprehensive search.

The databases were consulted between December 2019 and January 2024, and only English-language articles were included. Studies written in languages other than English were excluded, along with articles focused exclusively on other coronaviruses such as severe acute respiratory syndrome (SARS) and Middle East respiratory syndrome (MERS). Additionally, only studies with free full-text availability were considered. The research was conducted independently by two researchers, and their findings were compared to ensure consistency and minimize selection bias. Any discrepancies between the researchers were discussed and resolved through consensus. Furthermore, a quality assessment was performed for validation studies on neurological manifestations in patients with COVID-19 and its vaccination (see Table 1). All authors actively participated in the research process, literature selection, and manuscript writing. The manuscript was written in compliance with TITAN guidelines^[12]^.

**Table 1:** Quality assessment for validation studies on neurological manifestations of COVID-19 and its vaccination

First author	Title	Did the review mention neurological manifestations in COVID-19?	Does the study or the review meet our inclusion/exclusion criteria?	Does the review meet our objectives?	Was the sample size adequate?	Were the study subjects and the setting described in detail?	Was the data analysis conducted with sufficient coverage of the identified sample?
Wu F	A new coronavirus associated with human respiratory disease in China	Yes	No	No	Yes	Yes	Yes
Zhu N	A Novel Coronavirus from Patients with Pneumonia in China	Yes	Not Available	No	Yes	Yes	Yes
Guan WJ	Clinical Characteristics of Coronavirus Disease 2019 in China	Yes	Yes	Yes	Yes	Yes	Yes
Mehta P	COVID-19: consider cytokine storm syndromes and immunosuppression	No	No	No	Yes	Yes	Yes
Ousseiran ZH	Neurological manifestations of COVID-19: a systematic review and detailed comprehension	Yes	Yes	Yes	Yes	Yes	Yes
Mao L	Neurologic Manifestations of Hospitalized Patients With Coronavirus Disease 2019 in Wuhan, China	Yes	No	Yes	Yes	Yes	Yes
Caroli A	Brain diffusion alterations in patients with COVID-19 pathology and neurological manifestations	Yes	Yes	Yes	Yes	Yes	Yes
Battaglini D	Long-term neurological symptoms after acute COVID-19 illness requiring hospitalization in adult patients: insights from the ISARIC-COVID-19 follow-up study	Yes	Yes	Yes	Yes	Yes	Yes
Yaamika H	Review of adverse events associated with COVID-19 vaccines, highlighting their frequencies and reported cases	Yes	Yes	Yes	Yes	Yes	Yes
Lechien JR	Olfactory and gustatory dysfunctions as a clinical presentation of mild-to-moderate forms of the coronavirus disease (COVID-19): a multicenter European study	Yes	Yes	Yes	Yes	Yes	Yes
Harapan BN	Neurological symptoms, manifestations, and complications associated with severe acute respiratory syndrome coronavirus 2 (SARS-CoV-2) and coronavirus disease 19 (COVID-19)	Yes	Yes	Yes	Yes	Yes	Yes
Benito-León J	Unseen scars: Unraveling the neurological manifestations of COVID-19	Yes	Yes	Yes	Yes	Yes	Yes
Doblan A	Cranial nerve involvement in COVID-19	Yes	Yes	Yes	Yes	Yes	Yes
Paradiso B	An update on neurological disorders post COVID-19 infection	Yes	Yes	Yes	Yes	Yes	Yes
Gomez F	COVID-19: a modern trigger for Guillain-Barre syndrome, myasthenia gravis, and small fiber neuropathy	Yes	Yes	Yes	Yes	Yes	Yes
Molaverdi G	Neurological complications after COVID-19: A narrative review	Yes	Yes	Yes	Yes	Yes	Yes
Hameed R	Neurological and Psychiatric Manifestations of Long COVID-19 and Their [^(18)^F]FDG PET Findings: A Review	Yes	Yes	Yes	Yes	Yes	Yes
Zirpe KG	Pathophysiological Mechanisms and Neurological Manifestations in COVID-19	Yes	Yes	Yes	Yes	Yes	Yes
Zubair AS	Neuropathogenesis and Neurologic Manifestations of the Coronaviruses in the Age of Coronavirus Disease 2019: A Review	Yes	Yes	Yes	Yes	Yes	Yes
Ahmad I	Neurological manifestations and complications of COVID-19: A literature review	Yes	Yes	Yes	Yes	Yes	Yes
Mehta Y	Cytokine Storm in Novel Coronavirus Disease (COVID-19): Expert Management Considerations	No	No	No	Yes	Yes	Yes
Alonso Castillo R	Neurological manifestations associated with COVID-19 vaccine	Yes	Yes	Yes	Yes	Yes	Yes
Chatterjee A	Neurological Complications Following COVID-19 Vaccination	Yes	Yes	Yes	Yes	Yes	Yes
Yang Y	Neurological Disorders following COVID-19 Vaccination	Yes	Yes	Yes	Yes	Yes	Yes
Chen F	The Impact of COVID-19 and Vaccine on the Human Nervous System	Yes	Yes	Yes	Yes	Yes	Yes
Beretta S	Incidence and Long-term Functional Outcome of Neurologic Disorders in Hospitalized Patients With COVID-19 Infected With Pre-Omicron Variants	Yes	Yes	Yes	Yes	Yes	Yes
Ravindra	Spectrum of neurological complications following COVID-19 Vaccination	Yes	Yes	Yes	Yes	Yes	Yes
Aparajita	Neurological Complications Following COVID-19 Vaccination	Yes	Yes	Yes	Yes	Yes	Yes
Zeinab	A review of the potential neurological adverse events of COVID-19 vaccines	Yes	Yes	Yes	Yes	Yes	Yes
Roya	A review of neurological side effect COVID-19 vaccination	Yes	Yes	Yes	Yes	Yes	Yes
Sara	Post COVID-19 Vaccination-Associated Neurological Complications	Yes	Yes	Yes	Yes	Yes	Yes
Hardeep	COVID-19 vaccination-associated myelitis	Yes	Yes	Yes	Yes	Yes	Yes
Miao	Guillain-Barre syndrome following COVID-19 vaccines: A review of literature	Yes	Yea	Yes	Yes	Yes	Yes
A B Hill	The Environment and Disease: Association or Causation?	Yes	Yes	Yes	Yes	Yes	Yes
Shivam	Neurological toll of COVID-19	Yes	Yes	Yes	Yes	Yes	Yes
Ying Yang	Neurological Disorders following COVID-19 Vaccination	Yes	Yes	Yes	Yes	Yes	Yes
Matthias	Cerebral venous sinus thrombosis after adenovirus-vectored COVID-19 vaccination: review of the neurological-neuroradiological procedure	Yes	Yes	Yes	Yes	Yes	Yes

## Results and discussion

### Global impact and spectrum of neurological manifestations

Since the beginning of the pandemic, there have been over 774 million confirmed cases and more than 7 million deaths worldwide^[13]^. The COVID-19 pandemic, which is caused by the rapid spread of the SARS-CoV-2 virus, has had a significant impact worldwide. Along with the well-known respiratory symptoms, there has been an increasing recognition of neurological manifestations in COVID-19 patients. These include problems with smell and taste, cranial nerves dysfunction, peripheral nerve disorders affecting multiple parts of the body, cerebrovascular conditions, and inflammation in the central nervous system^[14]^. Neurological symptoms can also occur as a potential manifestation of long COVID-19 or post-COVID-19 syndrome (medically unexplained persistent symptoms after COVID-19 infection). Data on specific types of symptoms and the prevalence of neurological long COVID-19 are still evolving. However, the main neurological symptoms of long COVID-19 included confusion, anosmia, ageusia, fatigue/malaise, muscle aches/joint pain, and seizures. Also, additional symptoms encompassed dizziness, erectile dysfunction, fainting/blackouts, headache, loss of sensation, muscle weakness, paresthesia, vision problems, speech difficulties, swallowing/chewing difficulties, balance problems, tinnitus, and tremors^[15]^. Therefore, understanding the neurological symptoms of long COVID-19 is crucial for allocating appropriate healthcare resources and effectively managing this global healthcare burden^[15]^.

### Prevalence and early clinical presentations

**Figure 1.**
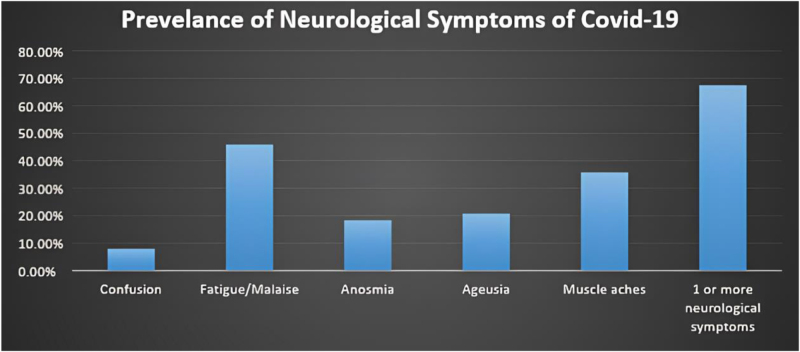
Observed prevalence and persistence of neurological symptoms over survey follow-up posthospital discharge.

Data were collected in recent studies based on the initial presentation of symptoms and whether those symptoms persisted. Participants without neurological symptoms upon hospitalization were classified under “first presentation,” while those with neurological symptoms evaluated during their initial hospitalization were categorized under “persistent presentation.” More than half of the participants had one or more neurological symptoms during their hospitalization^[15,16]^ (see Fig. 1). Additionally, another study highlighted various neurological issues of concern, including stroke, cerebral venous sinus thrombosis, meningoencephalitis, GBS, Miller Fisher syndrome, acute myelitis, and posterior reversible encephalopathy syndrome (PRES), systematically addressing these complications^[17]^. These studies emphasize the significant influence of the virus on the nervous system, both during the acute phase of infection and in cases of long COVID, where symptoms persist for prolonged periods even after the initial recovery^[18]^.

### Cranial nerve involvement

Several cranial nerves have been reported to be affected by COVID-19 infection, with clinical evidence indicating involvement of the 1st, 6th, 7th, 8th, 9th, 10th, and 12th cranial nerves. Among these, the effect on the olfactory nerve can lead to symptoms such as loss of smell (anosmia) and taste (ageusia); these symptoms may appear early as isolated presentations or before the respiratory manifestations^[16,19]^. Symptoms arising from involvement of other cranial nerves include double vision (diplopia), facial paralysis, hearing impairment (deafness), difficulty swallowing (dysphagia), and bulbar symptoms. These manifestations can arise from COVID-19 induced encephalitis or meningitis.

### Other common neurological manifestations and prognosis

Encephalopathy, acute cerebrovascular disease, and acute polyradiculopathy or neuropathies have been frequently reported in previous studies as the most common neurological complications associated with COVID-19, particularly among hospitalized patients. These manifestations have been linked to poorer clinical outcomes^[20,21]^. Other studies have identified GBS and its variants as the most frequently reported neurological manifestation (24%), followed by encephalopathy (21%)^[22,23]^. Nevertheless, the pathophysiological mechanism of GBS in patients with recent COVID-19 infection appears to be similar to that observed in GBS following other infectious triggers. Notably, the prevalence of central and peripheral nervous system involvement varies across different age groups^[24]^.

### Neurological manifestations in Long COVID-19

Over more than 2 years, extensive research has been dedicated to investigating the long-lasting effects of COVID-19, commonly referred to as “long COVID-19.” Studies have indicated that around 10% of individuals who have contracted COVID-19 continue to experience persistent symptoms. Of particular concern are the neurological and psychiatric manifestations associated with long COVID-19. Although these manifestations’ exact mechanisms are not yet fully understood, recent imaging studies have provided valuable insights into certain pathological aspects. Specifically, [18 F] FDG PET imaging has allowed researchers to directly examine cellular glycolysis, which is often associated with metabolic and inflammatory processes^[25]^.

### Neuroinvasion pathways

SARS-CoV-2 virus may affect the central nervous system (CNS) through several proposed mechanisms, including retrograde transsynaptic spread from infected neurons, entry via the olfactory nerve, transendothelial passage, and infiltration by infected leukocytes crossing the blood–brain barrier (BBB) via a “Trojan horse” mechanism. Damage to the olfactory epithelium may explain early sensory symptoms such as anosmia and ageusia. Systemic inflammation during infection can further increase BBB permeability, facilitating viral entry into the CNS^[26,27]^. Human coronaviruses have been detected in brain tissue, and accumulating evidence suggests potential long-term neurological consequences and chronic disease associations with COVID-19. Several studies have investigated these neuroinvasive pathways and their role in neuronal injury^[28,29]^.

### Mechanisms of neurological injury

Multiple studies show that neurological injury in SARS-CoV-2 infection is thought to arise from various mechanisms, primarily including hypoxic brain injury, immune-mediated damage, and cerebrovascular injury. Pneumonia associated with severe COVID-19 leads to respiratory insufficiency and subsequent hypoxia, which can result in chronic hypoxic brain injury. Immune-mediated brain injury occurs due to a cytokine storm, characterized by excessive release of inflammatory cytokines and activation of immune cells, leading to vascular leakage, complement activation, coagulation cascade dysregulation, and multi-organ failure. Cerebrovascular injury is attributed to SARS-CoV-2 binding to endothelial ACE-2 receptors, causing increased luminal pressure and potential brain bleeding, exacerbated by coagulation abnormalities. Additionally, potential mechanisms such as viral elimination by cytotoxic T cells, neuronal apoptosis as a protective response, and the unique homeostasis characteristics of CNS cells contribute to the overall pathology^[27,30,31]^.

### Overview of postvaccination neurological manifestations

The rapid spread of COVID-19 has led to a global pandemic, resulting in substantial illness and death. In response, numerous vaccines have been developed to combat this disease^[32]^. Neurological complications following COVID-19 vaccination are generally mild and transient, including headaches, fatigue, muscle, and joint pains. These mild effects are common with all types of COVID-19 vaccines. Severe neurological adverse events, such as GBS, seizures, syncope, encephalitis, Bell’s palsy, and strokes, are rare but have been reported^[33]^. Cerebral vascular events, including cerebral venous sinus thrombosis and arterial ischemic stroke, and the recently discovered AstraZeneca-induced thrombocytosis, have been associated with certain COVID-19 vaccines, particularly adenoviral vector vaccines^[34–36]^. The mechanism behind severe neurological complications such as cerebral venous sinus thrombosis is thought to be related to adenoviral vectored vaccine-induced thrombotic thrombocytopenia, where antibodies react with platelet factor 4, leading to hypercoagulability^[37,38]^. Other neurological complications reported postvaccination include acute disseminated encephalomyelitis, encephalopathy, myelitis, and neuroleptic malignant syndrome^[39–41]^.

While COVID-19 vaccination has been associated with rare postvaccination neurological adverse events, the overall incidence of such complications remains low. Compared to the neurological sequelae associated with SARS-CoV-2 infection, the risk posed by vaccination is significantly lower, supporting its widespread use as a preventive measure^[42]^. Following the introduction of mass vaccination programs, a decline in the overall incidence of COVID-19-related neurological disorders has been observed. Among individuals who experienced neuro-COVID manifestations, long-term functional outcomes were generally favorable, although mild symptoms have been reported to persist beyond 6 months in a subset of cases^[43]^. Current evidence continues to support vaccination, as the benefits outweigh the potential risks, and timely management is associated with improved outcomes in most cases of neurological involvement^[32,44,45]^.

### Pathophysiology

**Figure 2.**
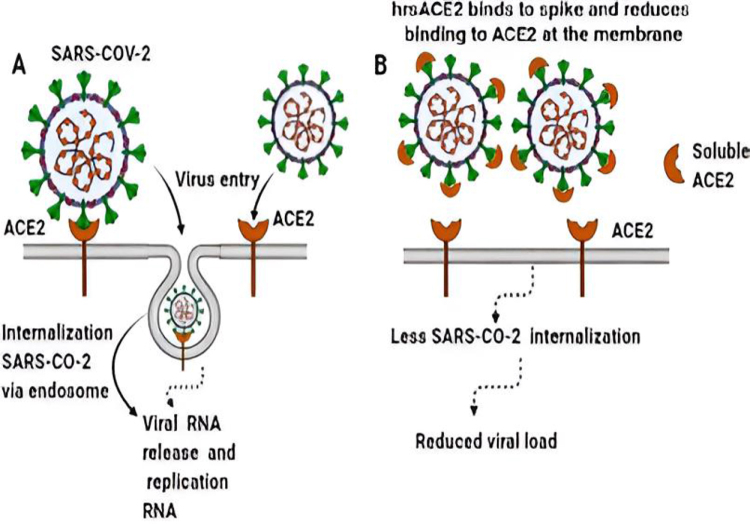
The invasion of SARS-COV2 by binding to the ACE2 receptors in the brain.

Evidence suggests that SARS-CoV-2 can breach the BBB and enter the brain. The virus may also travel hematogenously^[46]^ or using inflammatory cells to enter the CNS (myeloid cell trafficking)^[47]^. Additionally, retrograde axonal transport through the olfactory, respiratory, and enteric nervous systems is possible. After infecting the nasal cells, the virus can invade the brain directly, possibly through the olfactory bulbs and rapidly extend to specific brain areas such as the thalamus and brainstem, causing inflammation and demyelination. This invasion could explain the associated disruptions in smell and taste. The increasing evidence shows that COVID-19 may gain access to the CNS through a synapse-connected route after invading peripheral nerve terminals of the respiratory network^[48]^. SARS-CoV-2 enters host cells by binding to angiotensin-converting enzyme 2 (ACE2) receptors on various tissues^[49]^. In the brain, ACE2 is mainly expressed within the brainstem, the region responsible for cardiac and vascular function, which includes the nucleus of the solitary tract and the paraventricular nuclei^[50]^. The invasion of SARS-CoV-2 by the interaction with the ACE-2 is based upon the “Anchor-Locker” mechanism, in which the β-sheet and loops play a vital role in the complete transition process. Loop 2 is involved in the recognition and binding of ACE-2 and acts as an “Anchor”; on the other hand, loop 3 stabilizes the whole structure and acts as a “Locker” in this complete mechanism. After SARS-CoV-2 and ACE-2 have been recognized, β-sheet 1 works to strengthen and improve the binding Fig. 2 ^[51]^. Once in the brain, SARS-CoV2 may infect neurons and glial cells expressing ACE-2 enzyme, leading to cell death^[52]^.

Infection with SARS-CoV has previously been shown to be associated with high levels of cytokines, including tumor necrosis factor-alpha (TNFα), interleukin (IL)-1β, IL-6, IL-12, and interferon-gamma (INFγ), a phenomenon known as “cytokine storm”^[6,30,53]^. This “cytokine storm” acts as a chemoattractant for neutrophils, CD4 helper T cells, and CD8 cytotoxic T cells^[54]^. These proinflammatory cytokines associated with storm cytokines could contribute to microglia overactivation and also contribute to BBB disruption, leading to neuronal loss and specific neuropsychiatric manifestations such as confusion, disorientation, and headache. In severe cases, encephalitis and vascular damage could lead to ischemic events in patients. The neuroimage analysis evidenced brain-induced dysfunctions and neuropathological manifestations by COVID-19^[55]^.

## Neurological manifestations associated with COVID-19 vaccination

### Cerebral venous sinus thrombosis

It is the most common and serious complication, with incidence varying among different studies. It is commonly presented with treatment-resistant headaches a few days postvaccination, sometimes accompanied by seizures, altered sensorium, and focal deficits. The median time to its occurrence is 9 days (range: 2–45 days)^[56]^. Affected patients are generally females of younger ages^[45]^. Cases have been reported among individuals with autoantibodies against platelet factor 4 and elevated D-dimer levels^[57]^. CVST involves blood clots in the dural venous sinuses, cerebral veins, or both^[51]^. AstraZeneca (ChAdOx1) and Johnson & Johnson (Ad26.COV2) vaccines, which use adenoviral vectors encoding the SARS-CoV-2 spike glycoprotein, have been associated with CVST^[58]^. Interactions between the vaccine and platelets or platelet factor-4 (PF4) may contribute to the development of vaccine-induced immune thrombotic thrombocytopenia (VITT), possibly due to free DNA in the vaccines binding to PF4 and triggering autoantibodies^[59]^.

The free DNA in the vaccine could bind to PF4 and trigger platelet-activating immunoglobulin G (IgG) antibodies. This complex then binds to the platelet FcRγIIA receptors and causes platelet activation and the formation of platelet microparticles, these microparticles initiate the formation of blood clots and induces a prothrombotic cascade, which consequently decreases platelet count and causes thrombocytopenia. Moreover, the reticuloendothelial system, particularly the spleen, removes the antibody-coated platelets and aggravates thrombocytopenia^[60]^.

### Headache

Headache is one of the most common adverse effects of COVID-19 vaccination. Several studies have reported headaches after the first and second vaccine doses^[61,62]^. A meta-analysis showed that headache has migraine-like features with pulsating quality, phonophobia, and photophobia in approximately one-third of the patients receiving COVID-19 vaccines^[63]^.

Post-vaccination headaches can be caused by stress, vascular spasm, and intracerebral or subarachnoid haemorrhage. Adenovirus-based and mRNA vaccines are more frequently associated with headaches^[64]^. The Centers for Disease Control and Prevention recommends managing post-vaccination headaches with acetaminophen and/or non-steroidal anti-inflammatory drugs (NSAIDs)^[65]^.

### Myelitis

Acute transverse myelitis (ATM) is a rare vaccine-related consequence, possibly due to polyclonal activation of B lymphocytes^[66]^. The phase 3 clinical trials of AstraZeneca reported a few cases of transverse myelitis^[51]^. Recombinant ChAdOX1 nCoV-19 vaccine, during the trial phase, had been associated with two instances of myelitis. Of these, one was found to have multiple sclerosis in the background while the other was referred to as a possibly related event^[67]^.

### Guillain–Barré syndrome

With the widespread use of COVID-19 vaccines worldwide, many cases of postvaccination GBS have been reported^[68]^. According to preliminary reports, 132 cases of GBS occurred after the administration of 13.2 million doses of the Johnson & Johnson vaccine in the United States and 227 cases of GBS occurred after the administration of 51 million doses of the AstraZeneca vaccine in Europe^[68,69]^. DNA vaccines use a single recombinant replication-deficient chimpanzee adenovirus vector (ChAdOx1) to enter the cell and encode the spike protein of the SARS-CoV-2 virus, stimulating antibody and T-cell production. In mRNA vaccines, the mRNA molecules are included in lipid nanoparticles that allow the fusion with cellular membranes of host cells and hence the mRNA is released in the cytoplasm, where it is translated to build the spike protein. Antibodies against the spike protein may cross-react with peripheral nerve components (gangliosides) potentially causing GBS. However, contrary to expectations, GBS cases that occur after COVID-19 vaccination have a low positive rate of anti-ganglioside antibodies (20%), which is significantly lower than that (80–90%) in other reported GBS cases, a discrepancy suggesting that gangliosides may not be the true antigenic target for GBS that emerges after COVID-19 vaccination^[68,70]^. Treatment usually includes IVIG and plasma exchange in severely ill patients, and the prognosis varies greatly^[71]^.

The recurrence risk of COVID-19 vaccination for patients with a GBS history is still uncertain, but some research results show the safety signal^[72]^. A study showed that only 3.5% of patients have relapsed after vaccination^[44]^. Patients with neurological findings should be enquired about recent vaccination history. It is of enormous importance, especially after the COVID-19 mRNA vaccine, which is newly introduced, as it might link to the development of a wider variety of neurological presentations. The risks of the vaccines are not fully understood; however, the benefits of the vaccines appear to outweigh the risks that might be encountered^[73]^.

### Stroke

Stroke has been reported post-COVID-19 vaccination^[74]^, including ischemic, and hemorrhagic strokes^[75]^. The exact mechanism is not well understood^[60]^. A systematic review study indicated that ischemic stroke after viral vector vaccination occurred predominantly after the Oxford-AstraZeneca vaccine (30 cases), with fewer cases linked to Pfizer-BioNTech (7 cases), Moderna (1 case), CoronaVac (3 cases), and Sinopharm (1 case) vaccines^[76]^. Hemorrhagic strokes are classified as due to subarachnoid haemorrhage or intracerebral haemorrhage^[77]^. The majority of hemorrhagic strokes after COVID-19 vaccination occur in the context of vaccine-induced immune thrombotic thrombocytopenia (VITT)^[78]^.

## Conclusion

Neurological manifestations have been increasingly recognized in COVID-19 patients, including problems with smell and taste, cranial nerve impairments, nerve disorders affecting multiple parts of the body, cerebrovascular issues, and brain dysfunction. These manifestations can also occur in long COVID-19 cases. COVID-19 vaccination may lead to mild and transient neurological complications such as headaches and fatigue. Severe neurological adverse events following vaccination, such as GBS, seizures, and strokes, are rare but have been reported. The overall incidence of severe neurological complications post-vaccination is low, and vaccination is still recommended due to the benefits outweighing the risks. Understanding and addressing neurological manifestations and complications related to COVID-19 and vaccination remain crucial for effective healthcare management.

## Limitations

- This review provides a detailed analysis of the neurological manifestations associated with COVID-19 and its vaccines, yet several limitations must be acknowledged. First, the inclusion of only English-language articles and freely accessible full-text studies may have introduced selection bias, excluding relevant research from non-English sources or behind paywalls. Additionally, the heterogeneity of the included studies – varying in design, population, and methodology – makes it difficult to draw uniform conclusions.
- The dynamic nature of the pandemic means that findings may quickly become outdated as new SARS-CoV-2 variants emerge and vaccination strategies evolve. Furthermore, the long-term neurological effects of both COVID-19 and its vaccines remain incompletely understood due to limited follow-up periods in many studies. While the review discusses rare but severe post-vaccination complications, such as GBS and CVST, establishing causality remains challenging, as most evidence comes from case reports and observational studies rather than controlled trials.
- Demographic variability in neurological outcomes is not thoroughly explored, and data from low-resource settings (e.g., Sudan) are sparse despite their inclusion in the discussion. The review primarily focuses on hospitalized patients and severe cases, potentially overlooking milder or subclinical neurological effects. Vaccine-specific risks, particularly for adenoviral vector vaccines, are highlighted, but comparative analyses across different vaccine platforms (e.g., mRNA vs. inactivated vaccines) are limited.
- Diagnostic inconsistencies, such as varying neuroimaging protocols and criteria for neurological symptoms, further complicate data interpretation. Confounding factors, including pre-existing neurological conditions and concurrent infections, are not always accounted for, which may skew reported associations. Publication bias may also inflate the perceived frequency of rare adverse events, as severe cases are more likely to be published than milder ones.
- Finally, as a narrative review, this study lacks quantitative synthesis (e.g., meta-analysis), limiting its ability to provide precise risk estimates. Future research should prioritize standardized methodologies, longer follow-up periods, and more inclusive population studies to better understand the neurological risks of COVID-19 and its vaccines, ensuring balanced risk-benefit assessments for public health decision-making.

## Data Availability

The data that supports the findings of this study are available with the corresponding author upon reasonable request.

## Research registration unique identifying number (UIN)

Name of the registry: Not required (because this was a review article); Unique Identifying number or registration ID: Not applicable (because this was a review article); Hyperlink to your specific registration (must be publicly accessible and will be checked).
